# Low but not high exercise systolic blood pressure is associated with long-term all-cause mortality

**DOI:** 10.1136/bmjsem-2021-001106

**Published:** 2021-06-07

**Authors:** Kristofer Hedman, Leonard A Kaminsky, Ahmad Sabbahi, Ross Arena, Jonathan Myers

**Affiliations:** 1 Department of Clinical Physiology in Linköping, and Department of Health, Medicine and Caring Sciences, Linköping University, Linköping, Sweden; 2 Fisher Institute of Health and Well-Being, Ball State University, Muncie, Indiana, USA; 3 Department of Physical Therapy, College of Applied Health Sciences, University of Illinois at Chicago, Chicago, Illinois, USA; 4 School of Physical Therapy, South College, Knoxville, Tennessee, USA; 5 Cardiology Division, VA Palo Alto Health Care System, Stanford University, Palo Alto, California, USA

**Keywords:** exercise testing, cardiovascular epidemiology, risk factor, exercise physiology

## Abstract

**Objectives:**

The risks associated with achieving a high peak systolic blood pressure (SBP) during clinical exercise testing remain controversial, although this issue has not been evaluated in relation to predicted SBP standards. This cohort study aimed to evaluate the long-term risk of all-cause mortality in males in relation to reference values of peak SBP and the increase in SBP during exercise from the Fitness Registry and the Importance of Exercise: A National Database (FRIEND).

**Methods:**

We followed 7164 males (mean age: 58.2±10.6 years) over 95 998 person-years of follow-up (mean 13.4±5.4 years), who performed a maximal treadmill exercise test at baseline. SBP was measured at rest and at peak exercise. Risk of all-cause mortality over 20 years (Cox regression) was determined in relation to reference percentiles of peak SBP and increase in SBP with exercise: <10th (low), 10th–90th, >90th (high) percentiles.

**Results:**

A high peak or a large increase in SBP with exercise was not associated with all-cause mortality. Subjects with a low peak SBP had a 20% higher unadjusted risk for all-cause death compared with those with a normal value (1.20 (1.11–1.31)), and a statistically non-significant 7% higher risk after adjustment for all baseline risk factors (1.07 (0.97–1.18)). The corresponding unadjusted and adjusted risks associated with a low increase in SBP were 1.24 (1.15–1.35) and 1.11 (1.02–1.21), respectively.

**Conclusions:**

A low—but not high—peak SBP is associated with increased unadjusted risk of all-cause mortality. The FRIEND percentiles of exercise SBP can aid clinicians in individualising risk assessment.

**Key messages**

**What is already known**

- Systolic blood pressure rises during progressive ramp exercise to maximal values affected by age, sex and exercise workload.
- Previous studies using arbitrary thresholds for exercise blood pressure have found both increased and decreased risk of mortality with an exaggerated systolic blood pressure with exercise.

**What are the new findings**

- This study evaluated the association between exercise systolic blood pressure and mortality, using published normative values for exercise systolic blood pressure, accounting for age and sex.
- We found that an exercise systolic blood pressure in the lower 10th percentile—but not in the upper 90th percentile—was associated with increased mortality.
- It is thus important to identify individuals with an inability to adequately increase systolic blood pressure during exercise, which could be facilitated by using reference standards.

## Introduction

A failure to significantly increase systolic blood pressure (SBP) above resting levels during an incremental exercise test, or a subsequent drop in SBP are both acknowledged markers of underlying cardiovascular disease and are associated with future mortality.^1–4^ The prognostic implications of reaching a high SBPpeak during the exercise testing is more ambiguous,^5 6^ and there have been reports of higher^7–9^ as well as lower^10–13^ risk of mortality in subjects with relatively greater increases in SBP during exercise. However, comparisons across studies are difficult in part due to a large variation in how an exaggerated SBP response to exercise is defined as well as in terms of variation in cardiovascular risk profiles of patients included.^14 15^

In the most recent scientific statement on exercise standards for testing and training from the American Heart Association (AHA) published in 2013,^16^ the threshold for defining an exaggerated SBPpeak response to exercise is ≥210 mm Hg in males and ≥190 mm Hg in females, regardless of age. The same thresholds are suggested by the American College of Sports Medicine (ACSM).^17^ More recently, age-specific and sex-specific reference standards for SBPpeak have been published for treadmill^18^ as well as for lower extremity ergometer^19^ exercise testing, suggesting a need to consider both sex and age in clinical SBPpeak interpretation.^20^ However, the prognostic implications of the SBP response to exercise in relation to reference standards has not been evaluated.

This study sought to examine the long-term risk of all-cause mortality in US male Veterans referred to clinical exercise testing, using recent reference percentiles for SBPpeak and the SBP increase from rest to peak exercise (ΔSBP), derived from the Fitness Registry and the Importance of Exercise National Database (FRIEND).^18^

## Methods

### Study design and cohort

From 9831 exercise tests performed at the Veterans Affairs (VA) Health Care System in Palo Alto, California between 1987 and 2007, we considered the 9079 (92.3%) tests performed by males on a treadmill. We excluded 758 subjects with missing data in terms of SBP at rest and/or peak exercise, exercise capacity (in metabolic equivalents (METs)), birth date, test date or date of death. We also excluded 422 subjects with any of the following pre-existing comorbidities: (1) heart failure; (2) congenital heart disease; (3) hypertrophic cardiomyopathy; (4) implanted pacemaker or cardiac defibrillator; (5) heart transplant or cardiac support device and (6) severe valvular heart disease. We also excluded the following subjects: (1) those with a follow-up time <26 weeks (n=102); (2) subjects with an SBP standing at rest <80 mm Hg or >250 mm Hg (n=19); (3) subjects reaching a peak MET value <3 (n=157) or a peak Borg rating of perceived exertion <15 in combination with <70% of age-predicted maximal heart rate (n=79); (4) those with a drop or no increase (0 mm Hg) in SBP from rest to peak exercise (n=244) and (5) subjects≥80 years, as the age span for the reference standards used in the current study was 20–79 years (n=134).^18^

From each subject’s computerised medical record, demographic, clinical and medication information was obtained prior to the exercise test. We ascertained vital status of the participants as of 20 July 2018. Date of death was verified using the VA Beneficiary Identification and Record Locator System File.^21^ Follow-up time was defined as the time from the exercise test to either date of death or the date when a subject was last verified to be alive.

### Patient and public involvement

This study was originally initiated in 1987, and patients or the public were not involved in planning of this study.

### Exercise and blood pressure assessments

Each subject underwent a standardised treadmill exercise test using an individualised ramp protocol with standard criteria for termination, including signs of inducible cardiac ischaemia or a sustained drop in SBP.^16^ An SBP >250 mm Hg or a diastolic BP >115 mm Hg were relative indications for test termination.^16^ Exercise capacity in METs was estimated using standard ACSM equations.^17^ Details on the VA exercise testing protocol used and the ACSM formulas have been previously published,^10^ and are presented in online supplemental methods S1.

10.1136/bmjsem-2021-001106.supp1 Supplementary data

Blood pressure was measured by auscultation before and during the treadmill test in accordance to current AHA guidelines for exercise testing laboratories.^22^ Before exercise, SBP was measured once in the right arm with the subject standing at rest on the treadmill (SBPrest). During the exercise, SBP was measured in the right arm every second to third minute, where the subject was instructed to let go of the handlebars and SBP was recorded at the appearance of the first Korotkoff sound. The last (peak) measurement was recorded just prior to test termination (SBPpeak). The difference between SBPrest and SBPpeak was defined as ΔSBP.

### Reference sample: FRIEND

Each subject’s SBPpeak and ΔSBP value were categorised using age-specific reference percentiles recently published from the FRIEND registry.^18^ Reference percentiles were determined from 1525 apparently healthy males between 20–79 years of age. In brief, FRIEND was launched in 2014 as an initiative of the AHA to standardise and collect exercise testing data from multiple laboratories across the United states.^23^ Blood pressure measurement in FRIEND was performed in accordance to the same guidelines as in the current study.^22^

The SBPpeak and ΔSBP values for each subject were categorised as: (1) ‘Low’ when falling below the 10th percentile in FRIEND; (2) ‘Normal’ when within the 10th–90th percentile and (3) ‘High’ when above the 90th percentile. As an online supplemental analysis, we also used the lower 5th and upper 95th percentiles for similar comparisons.

### Statistical analysis

We used SPSS software V.26.0 (IBM) for database management and statistical analysis. Survival analysis was performed using R Studio V.1.1.456 (R Studio, Vienna, Austria) with the survival (V.2.38) and survminer (V.0.4.3) packages. Continuous variables were presented as mean (SD), using one-way ANOVA or the χ^2^ test to determine statistically significant differences between groups. Two-sided statistical significance was set at <0.05.

Outcome analyses were performed with 20-year all-cause mortality as the endpoint. The Kaplan-Meier method with the log-rank (Mantel-Cox) method was used to compare survival per FRIEND reference percentile of the variables of interest. Unadjusted and adjusted Cox proportional HRs were calculated. Natural cubic spline modelling was used to characterise the risk associated with SBPpeak and ΔSBP as continuous variables, using three knots placed at 25th, 50th and 75th percentiles. The adjusted models included (a) age, (b) plus SBPrest and exercise capacity (in METs), (c) plus baseline cardiovascular risk factors (ie, diabetes mellitus, hyperlipidaemia, hypertension, body mass index, current smoking and previous (known) coronary artery disease). Use of antihypertensive medication and statin use were included in the definition of hypertension and hyperlipidaemia, respectively. Details on how each covariate was defined is provided in online supplemental methods S2.

## Results

**Figure 1:**
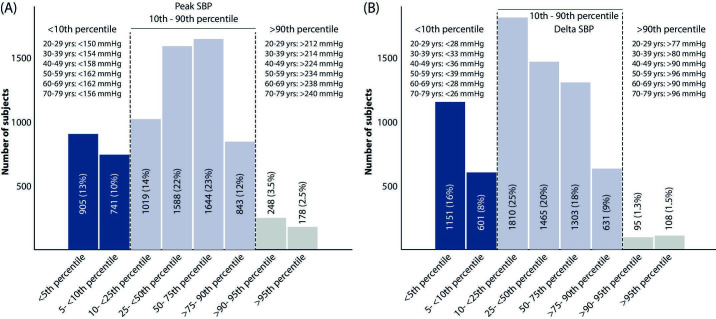
Number of subjects in the current cohort of male veterans falling into each of the eight categories of reference percentiles suggested in reference cohort (FRIEND). (A) SBPpeak with age-specific thresholds defining lower 10th and upper 90th percentiles; (B) delta SBP from rest to SBPpeak with age-specific thresholds defining lower 10th and upper 90th percentiles. Reference values from FRIEND published in Sabbahi *et al*.^18^ FRIEND, Fitness Registry and the Importance of Exercise: A National Database; SBP, systolic blood pressure.

In total, 7164 males between 21 and 79 years of age (58.2±10.6 years) were included. Of these, 23% had a low and 6% had a high SBPpeak, according to the reference percentiles, while 25% had a low and 3% had a large ΔSBP from rest to peak exercise (figure 1).

There was a difference in both SBP and DBP at rest (standing) between SBPpeak categories (both p<0.001), with successively greater values from subjects with low to normal and high SBPpeak groups (table 1). One-third of subjects with a low peakSBP had a history of coronary artery disease, as compared with 20% and 12% in subjects with a normal or a high peakSBP, respectively (p<0.001).

**Table 1:** Subject characteristics at time of exercise test, per peak systolic blood pressure reference category

	Low SBPpeak (<10 th)	Normal SBPpeak (10th −90th)	High SBPpeak (>90 th)	P value*
No. subjects (%)	1644 (23)	5094 (71)	426 (6)	–
Age, years	57.5±9.5	58.6±10.8	55.1±11.4	<0.001
Height, m	1.76±0.08	1.76±0.08	1.77±0.08	0.026
BMI, kg/m^2^	27.9±5.2	28.7±5.1	29.7±5.2	<0.001
METs	7.8±3.1	9.0±3.4	9.9±3.6	<0.001
SBPrest, mm Hg	118±13	135±18	149±19	<0.001
DBPrest, mm Hg	76±10	83±10	89±11	<0.001
SBPpeak, mm Hg	147±12	187±16	232±13	<0.001
Delta SBP, mm Hg	29±13	52±19	83±20	<0.001
SBP/MET-slope, mm Hg/MET	5.1±3.3	7.6±4.4	11.5±6.7	<0.001
Smoking, n (%)	880 (54)	2509 (49)	201 (47)	0.005
Diabetes mellitus, n (%)	217 (13)	718 (14)	71 (17)	0.095
Hypertension, n (%)	864 (53)	3050 (60)	282 (66)	<0.001
Previous CAD, n (%)	542 (33)	1008 (20)	53 (12)	<0.001
Hyperlipidaemia, n (%)	606 (37)	1984 (39)	168 (39)	0.29
Beta-blocker medication, n (%)	507 (31)	902 (18)	49 (12)	<0.001
Stroke, n (%)	52 (3)	133 (3)	10 (2)	0.43
Claudication, n (%)	61 (4)	200 (4)	9 (2)	0.17
COPD, n (%)	85 (5)	230 (5)	11 (3)	0.07

*P value for statistical comparison across all three groups with one-way ANOVA (means) or χ2 test (proportions). Reference values from FRIEND (Fitness Registry and the Importance of Exercise: A National Database) published in Sabbahi *et al*.^18^

ANOVA, analysis of variance; BMI, body mass index; CAD, coronary artery disease; COPD, chronic obstructive pulmonary disease; DBP, diastolic blood pressure; MET, metabolic equivalent; SBP, systolic blood pressure.

### Outcome analysis

**Figure 2:**
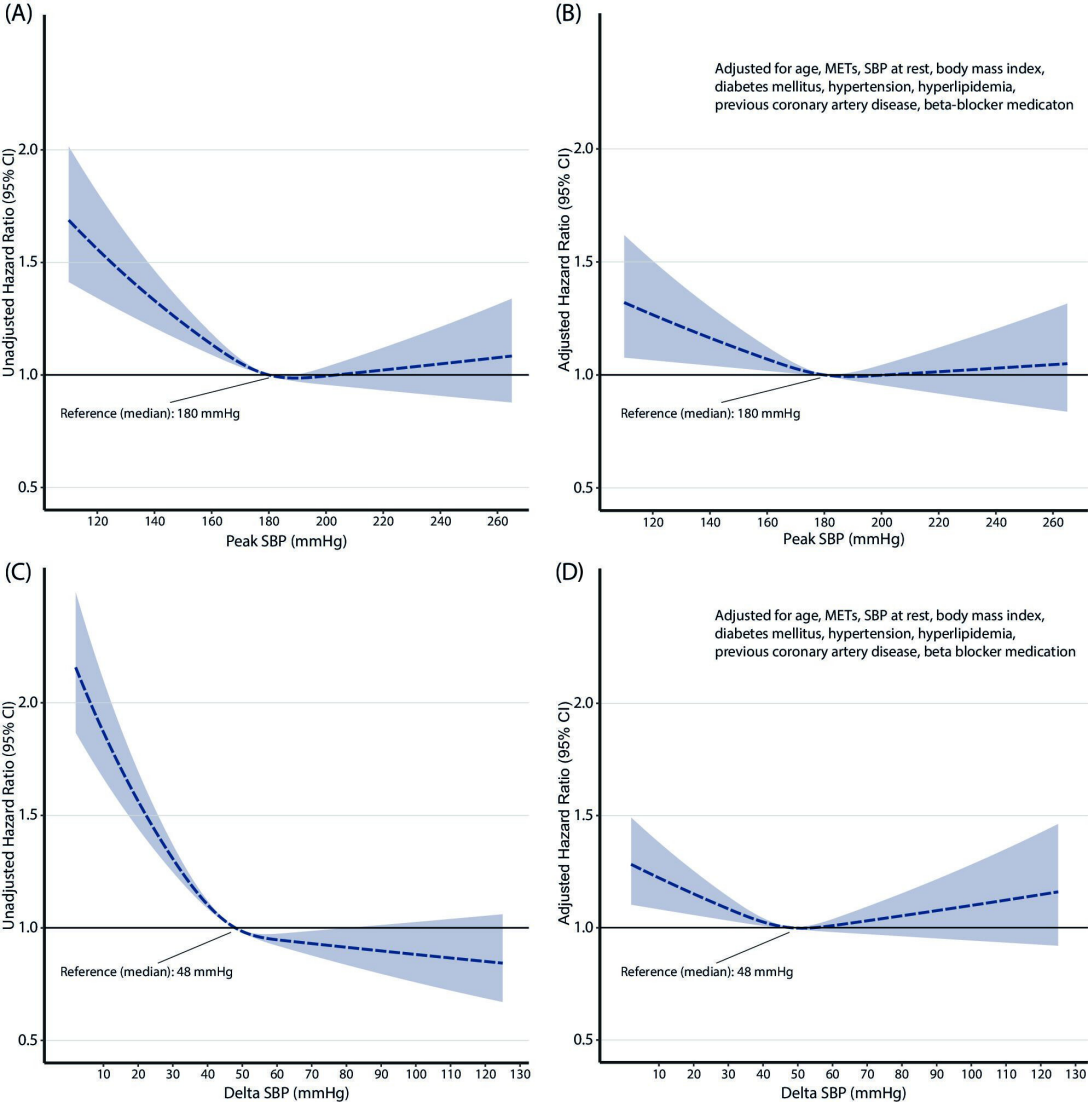
Continuous relative risk of all-cause mortality over 20 years per peak systolic blood pressure (SBP) (A, B) and increase in SBP (C, D) during treadmill exercise testing. A and C present unadjusted risk (HR with 95% CI); B and D adjusted for age, exercise capacity (metabolic equivalent of task (METs)), SBP at rest (standing), body mass index, a diagnosis of diabetes mellitus, hypertension, hyperlipidaemia, coronary artery disease and beta blocker medication.

During a mean follow-up of 13.4±5.4 years (truncated at 20 years), 3034 (42%) of subjects died (95 998 person-years of follow-up; 31.6 deaths per person-year). The relative risk of dying increased continuously with lower absolute values of peakSBP and ΔSBP, in both unadjusted and adjusted analyses (figure 2). There was no statistically significant increase in risk of all-cause mortality associated with having an SBPpeak or ΔSBP value higher than the overall median value (peakSBP: 180 mm Hg; ΔSBP: 48 mm Hg).

#### Reference categories

**Figure 3:**
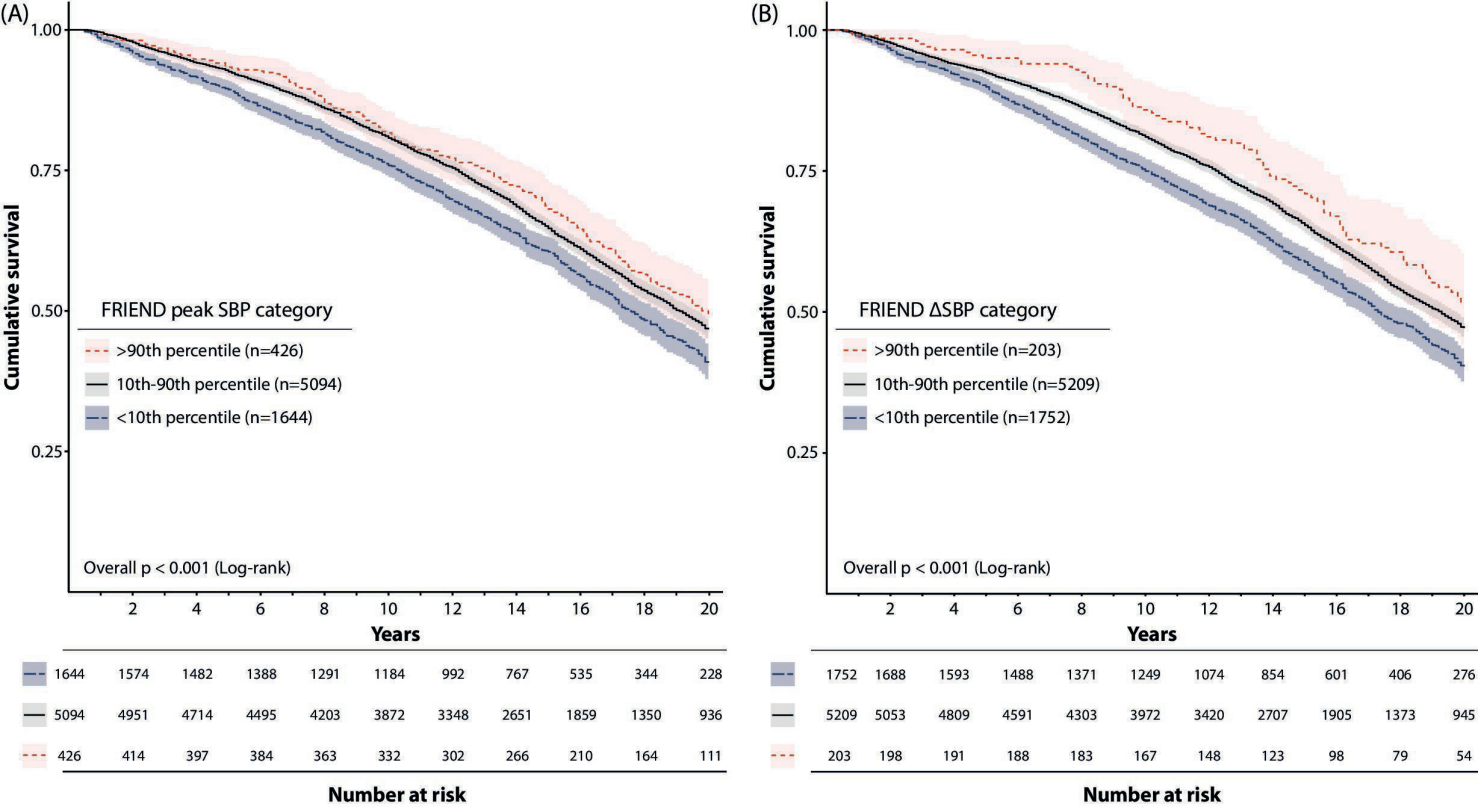
Cumulative crude survival per reference category of peak systolic blood pressure (SBP) and increase in systolic blood pressure, respectively. Subjects with a peak SBP(A) or an increase in systolic blood pressure with exercise (ΔSBP, B) falling in the lower age-specific 10th percentile (blue) had lower survival than subjects within the 10th–90th (grey) and upper 90th percentile (light red), respectively. Shaded area represents 95% CI. Reference values from FRIEND published in Sabbahi *et al*.^18^ FRIEND, Fitness Registry and the Importance of Exercise: A National Database.

As seen in figure 3, survival was lower for subjects in the lower 10th percentile of peakSBP (figure 3A) as well as ΔSBP (figure 3B). In addition, there was a trend for higher survival in subjects in the upper 90th percentile of ΔSBP.

Being in the lower 10th percentile reference category of SBPpeak was associated with a 14% higher risk of all-cause mortality (1.14 (1.04–1.25)) after adjusting for age, exercise capacity and SBPrest. When also adjusted for baseline risk factors and medication, the risk was no longer statistically significant (1.07 (0.97–1.18)). For ΔSBP, a similar association of higher risk with lower ΔSBP from rest to peak exercise was seen, although low ΔSBP was still significantly associated with higher mortality after full adjustment: 1.11 (1.02–1.21). Of note, high peakSBP or high ΔSBP were not associated with risk of all-cause mortality in unadjusted or adjusted analyses. Similar results (but with wider CIs) were seen when applying the 5th and 95th reference percentiles from FRIEND (online supplemental table S1).

#### Comparison with the AHA threshold

In total, 1139 males had an exercise SBPpeak ≥210 mm Hg (ie, exaggerated SBP response according to the AHA). Of these, 714 subjects (63%) were categorised as within the 10th–90th percentile according to FRIEND, while 425 (37%) exceeded the upper 90th percentile. In contrast to peakSBP (figure 2), there was no difference in survival between subjects below or above the AHA threshold (≥210 mm Hg) defining an exaggerated SBP response to exercise (p=0.1, (online supplemental figure S1). The relative risk of all-cause mortality for subjects exceeding the AHA threshold was similar to subjects below the threshold in unadjusted (0.92 (0.84–1.02)) and fully adjusted analyses: 0.97 (0.87–1.08), with the same covariates applied as in model 3 in table 2.

**Table 2:** Relative risk of all-cause mortality over 20 years per peak SBP and delta SBP reference category, respectively

	Unadjusted	Model 1	Model 2	Model 3
	Adjusted for			
	–	Age	Age, exercise capacity, SBP at rest	Model 2 plus risk factors* and beta-blockers
Peak systolic blood pressure category				
<10th percentile (n=1644)	**1.20 (1.11–1.31)**	**1.34 (1.24–1.46)**	**1.14 (1.04–1.25)**	1.07 (0.97–1.18)
10th–90th percentile (n=5094)	Reference	Reference	Reference	Reference
>90th percentile (n=426)	0.91 (0.78–1.06)	1.09 (0.93–1.26)	1.13 (0.97–1.32)	1.12 (0.96–1.31)
Delta systolic blood pressure category				
<10th percentile (n=1752)	**1.24 (1.15–1.35)**	**1.36 (1.25–1.47)**	**1.16 (1.07–1.26)**	**1.11 (1.02–1.21)**
10th–90th percentile (n=5209)	Reference	Reference	Reference	Reference
>90th percentile (n=203)	0.84 (0.67–1.05)	1.04 (0.83–1.30)	1.13 (0.90–1.41)	1.15 (0.92–1.44)

Bold font style denotes a statistically significant hazard ratio.

*Risk factors include body mass index, current smoking, diabetes mellitus, hypertension, hyperlipidaemia or a previous diagnosis of coronary artery disease. Model 3 also adjusted for use of beta-blocker medication. Reference values from FRIEND (Fitness Registry and the Importance of Exercise: A National Database) published in Sabbahi *et al*.^18^

SBP, systolic blood pressure.

## Discussion

To our knowledge, the current study is the first to evaluate the prognostic value of exercise SBP in relation to reference standards. We found that both a low SBPpeak and a small ΔSBP from baseline to peak exercise (in the lower 10th reference percentile) were associated with increased risk of long-term all-cause mortality. In contrast, a comparatively high SBPpeak or a large ΔSBP did not infer a greater risk of death during the follow-up.

Repeated measurements of SBP before, during and following exercise are an integral part of clinical exercise testing.^16 17 22^ A sustained drop in SBP >10 mm Hg in SBP with an increase in workload is considered an absolute or relative criterion for test termination, depending on whether other signs of cardiac ischaemia are present, and an SBP during exercise >250 mm Hg is a relative criterion for test termination.^16 17^ However, little guidance is offered for clinicians in terms of interpreting the SBP response to exercise in the absence of any adverse SBP events. Recently, the first update on reference standards for SBPpeak and ΔSBP for over 20 years was published,^18^ derived from FRIEND. We applied the age-specific and sex-specific 10th and 90th percentiles from these standards to define groups with low, normal or high SBPpeak and ΔSBP, which we believe could offer a novel, meaningful way of categorising each patient’s SBP response and help in test interpretation.

The negative prognostic implications of a drop in SBP or a failure to increase SBP above resting levels during exercise are well established.^1–4^ In contrast, the risk associated with high SBPpeak is more controversial.^5 6^ Studies report higher^7–9^ and lower^10–13^ risk of mortality in subjects with greater SBPpeak during exercise. Differences in patient populations, including in baseline cardiovascular risk profiles and age likely explain part of the discrepancy in previous results. When considering age and sex through the use of reference percentiles, we found no significant increase in risk of all-cause mortality for either SBPpeak achieved or ΔSBP. In our model adjusted for age, SBP at rest, exercise capacity and several cardiovascular risk factors (figure 2B), there was no increase in mortality observed with any SBPpeak values above the median, when plotted against SBPpeak on a continuous scale. In contrast, having a low SBPpeak or a low ΔSBP was associated with 20% and 24% higher unadjusted risks of all-cause mortality, respectively, compared with values within the 10th and 90th percentiles. Of note, even after adjusting for age, sex, SBP at rest and exercise capacity, there were still 14% and 16% higher risks of all-cause mortality in these groups. After additional adjustment for baseline cardiovascular risk factors and use of beta-blockers, there was a statistically non-significant 7% higher risk in subjects with a low SBPpeak, and an 11% statistically significant higher risk among subjects with high ΔSBP. Thus, baseline risk factors could explain some, but not all of the increase in risk associated with a blunted SBP response at exercise.

In this context, at a minimum, age and sex appear to be important considerations when analysing future risk of events in relation to SBP. As exercise SBP is related to exercise intensity through cardiac output, incorporating exercise intensity or exercise capacity in either reference equations for SBPpeak or in the outcome analysis is important. Recent European reference equations for bicycle ergometry suggest including sex, age, SBP at rest and exercise workload (in Watts) for predicting SBPpeak.^19^ The threshold in the current AHA guidelines for exercise testing to define an exaggerated SBP response to exercise (ie, ≥210 mm Hg in males) consider sex, but not age.^16^ Using this threshold could not predict future all-cause mortality in the current study, possibly due to a lack of precision when not considering the age dependency of SBPpeak.

The combined effect of poor exercise capacity and low SBPpeak has previously been shown to be particularly negative for prognosis.^24^ Hedman *et al*^10^ and Currie *et al*^6^ have suggested an alternative approach to consider workload in exercise SBP evaluation; the SBP/MET-slope. This measure index ΔSBP to the increase in METs from rest to peak exercise, and the relation between SBP and the SBP/MET-slope has recently been described in healthy athletes.^25^ This measure accounts for the confounding effect of workload, associated with both the risk of mortality and exercise SBP. In contrast to our previous finding of higher risk of all-cause mortality in subjects with higher SBP/MET-slope,^10^ subjects in the lower 10h percentile (with the highest risk of all-cause mortality in the current study) presented with the lowest mean SBP/MET-slope. These conflicting results may in part be explained by an association between hypertension and the SBP/MET-slope, or the lack of age and sex specific normative values of the SBP/MET-slope, as in terms of peak SBP. Future research should determine if the SBP/MET-slope or SBPpeak provides greater prognostic clarity than peak SBP, when considering age and sex.

### Limitations

First, this study only included male subjects, due to a very small proportion of females in the overall cohort, and further studies including females are required. Second, we lacked data on incident hypertension, cardiovascular disease and cardiovascular specific death which could add further insight in the prognostic implications of the SBP response to exercise. Third, given that the cohort represents clinical referrals, the results may not necessarily apply to a general population. Finally, the study included subjects over a span of 20 years, and medications as well as subject characteristics may have changed during the study period.

## Conclusion

Published reference percentiles for SBPpeak and ΔSBP predict long-term all-cause mortality in males, independent of age, exercise capacity, SBP at rest and age. Lower than predicted SBPpeak or a limited increase in SBP with exercise should be considered negative prognostic markers and could reflect underlying cardiovascular disease requiring additional follow-up.

## Data Availability

The data that support the findings of this study are available on reasonable request from the corresponding author, KH.
