# Supplementary material for: Low but not high exercise systolic blood pressure is associated with long-term all-cause mortality
Source: BMJ Open Sport Exerc Med. 2021 Jun 7;7(2):e001106. doi: 10.1136/bmjsem-2021-001106 (PMC8190063; doi:10.1136/bmjsem-2021-001106)
Supplement: Supplementary data [file bmjsem-2021-001106supp001.pdf]

DATA SUPPLEMENT

Low But Not High Exercise Systolic Blood Pressure is Associated with Long-Term All-Cause Mortality

Supplementary data

Kristofer Hedman, Leonard A Kaminsky, Ahmad Sabbahi, Ross Arena, Jonathan Myers

1. Supplementary Methods

S1. Details on exercise testing and exercise capacity estimation 2
S2. Definitions of cardiovascular comorbidities and risk factors 4

2. Supplementary Tables

Table S1. Relative risk of all-cause mortality over 20 years per 5th /95th percentiles 5

3. Supplementary Figures

Figure S1. Survival per FRIEND categories and AHA threshold 6

Hedman K, *et al. BMJ Open Sp Ex Med* 2022; 7:e001106. doi: 10.1136/bmjsem-2021-001106

DATA SUPPLEMENT

Supplementary methods S1.

Details on exercise testing and exercise capacity estimation

Each subject underwent a standardized treadmill exercise test using an individualized ramp protocol. The choice of protocol was individualized based on estimated exercise capacity and a targeted 8-12 min exercise duration, using the one-page Veterans Specific Activity Questionnaire. Subjects were encouraged to exercise without handrail support until volitional fatigue in absence of any indication for stopping the test (see below). Age-predicted maximal heart rate was not used as a target endpoint. The degree of effort was quantified using the Borg scale of perceived exertion.

Standard criteria for termination were used, including signs of inducible cardiac ischemia (i.e >2.0 mm horizontal/downsloping ST depression, moderately severe angina, a sustained drop in systolic blood pressure (SBP) or serious rhythm disturbances). A SBP >250 mmHg or a diastolic BP >115 mmHg were relative indications for test termination.

Peak exercise capacity was estimated as metabolic equivalents of task (METs), which is a surrogate measure of peak oxygen uptake, expressed as multiples of an assumed oxygen uptake (VO_2) at rest of 3.5 mL/kg/min. Thus, a peak exercise capacity of 10 METs corresponds to an estimated peak VO_2 of 35 mL/kg/min. Standard American College of Sports Medicine (ACSM) equations were used for calculating peak METs:

a) Walking $VO_2 = (S \times 0.1) + (S \times G \times 1.8) + 3.5$
b) Running $VO_2 = (S \times 0.2) + (S \times G \times 0.9) + 3.5$

where S is treadmill speed (in m/min) and G is grade (%, in decimal form). A treadmill speed >5.0 miles per hour (>134 m/min) were used to define running vs. walking VO_2.

Age-predicted peak METs was calculated using a population-specific equation as: $18 - [0.15 \times \text{Age}]$.

DATA SUPPLEMENT

Supplementary Methods S2.

Definitions of cardiovascular comorbidities and risk factors

We used the following definitions in determining the presence of cardiovascular comorbidities and risk factors at baseline exercise testing:

- **Previous coronary artery disease (CAD)**; previous myocardial infarction, cardiac procedures/interventions and/or coronary artery stenosis >50% at imaging.
- **Hypertension**; previous diagnosis of hypertension and/or use of any anti-hypertensive medication (not including beta-blockers).
- **Smoking**; Current smoker or previous smoker with history of >10 pack-years of smoking.
- **Hypercholesterolemia**; Total cholesterol >220 mg/dL, statin use, or both.
- **Diabetes mellitus**; a diagnosis at time of test per medical records.

Hedman K, *et al. BMJ Open Sp Ex Med* 2022; 7:e001106. doi: 10.1136/bmjsem-2021-001106

DATA SUPPLEMENT

Table S1. Relative risk of all-cause mortality over 20 years per 5th and 95th reference percentiles for peak systolic blood pressure and delta systolic blood pressure, respectively.

	**Unadjusted**	**Model 1**	**Model 2**	**Model 3**
	Adjusted for			
	-	**Age**	**Age, exercise capacity, SBP at rest**	**As model 2 plus risk factors* and beta-blocker med**
Peak systolic blood pressure category				
<5th percentile (n=903)	**1.23** (1.11-1.36)	**1.40** (1.27-1.56)	**1.14** (1.02-1.28)	1.04 (0.92-1.16)
5-95th percentile (n=6083)	Reference	Reference	Reference	Reference
>95th percentile (n=178)	**0.75** (0.59-0.96)	1.09 (0.85-1.39)	1.13 (0.88-1.45)	1.21 (0.94-1.57)
Delta systolic blood pressure category				
<5th percentile (n=1151)	**1.29** (1.18-1.42)	**1.42** (1.29-1.55)	**1.20** (1.09-1.32)	**1.14** (1.03-1.25)
5-95th percentile (n=5905)	Reference	Reference	Reference	Reference
>95th percentile (n=108)	**0.67** (0.48-0.93)	0.86 (0.62-1.20)	0.96 (0.69-1.34)	1.01 (0.73-1.41)

*, risk factors include body mass index, current smoking, diabetes mellitus, hypertension, hyperlipidemia or a previous diagnosis of coronary artery disease. Model 3 also adjusted for use of beta-blocker medication. SBP, systolic blood pressure. Reference values from FRIEND (Fitness Registry and the Importance of Exercise: A National Database) published in Sabbahi et al. Hypertension. 2018;71:229-236. DOI: 10.1161/HYPERTENSIONAHA.117.10116.

Hedman K, *et al. BMJ Open Sp Ex Med* 2022; 7:e001106. doi: 10.1136/bmjsem-2021-001106

Supplemental material BMJ Publishing Group Limited (BMJ) disclaims all liability and responsibility arising from any reliance placed on this supplemental material which has been supplied by the author(s) *BMJ Open Sp Ex Med*

DATA SUPPLEMENT

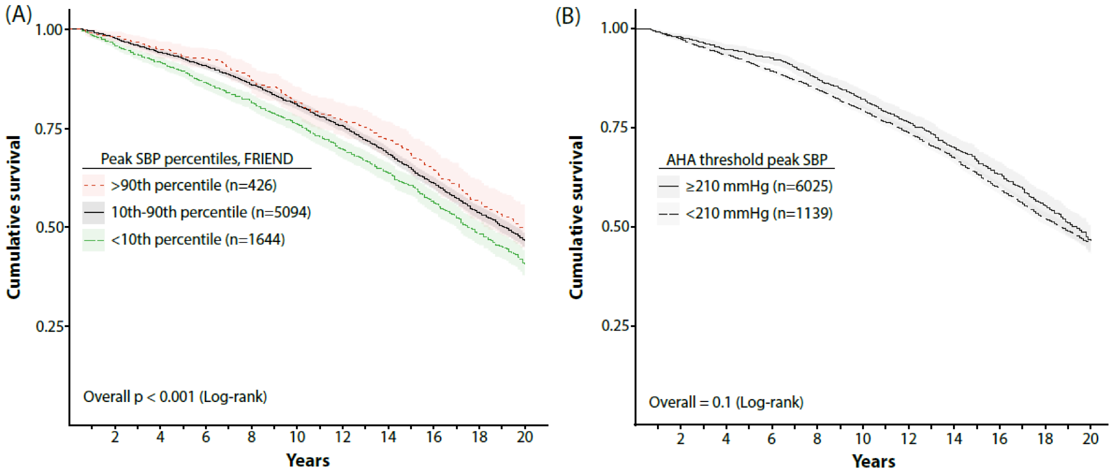

Figure S1. Survival over 20-years per peak systolic blood pressure categorized by FRIEND reference percentiles (panel A) or the AHA threshold (B).
AHA, The American Heart Association; SBP, systolic blood pressure.
Reference categories in panel A from FRIEND (Fitness Registry and the Importance of Exercise: A National Database) published in Sabbahi et al. Hypertension. 2018;71:229-236. DOI:10.1161/HYPERTENSIONAHA.117.10116. The AHA threshold from Exercise Standards for Testing and Training: A Scientific Statement From the American Heart Association, published by in Fletcher et al. Circulation. 2013; 128:873-934.
